# Vicarious Group-Based Rejection: Creating a Potentially Dangerous Mix of Humiliation, Powerlessness, and Anger

**DOI:** 10.1371/journal.pone.0095421

**Published:** 2014-04-23

**Authors:** Tinka M. Veldhuis, Ernestine H. Gordijn, René Veenstra, Siegwart Lindenberg

**Affiliations:** 1 Department of Sociology, University of Groningen, Groningen, The Netherlands; 2 Interuniversity Center for Social Science Theory and Methodology (ICS), University of Groningen, Groningen, The Netherlands; 3 Department of Social Psychology, University of Groningen, Groningen, The Netherlands; 4 Tilburg Center for Behavioral Economics (TIBER), Tilburg University, Tilburg, The Netherlands; Brock University, Canada

## Abstract

Rejection can convey that one is seen as inferior and not worth bothering with. Is it possible for people to feel *vicariously* rejected in this sense and have reactions that are similar to those following personal rejection, such as feeling humiliated, powerless, and angry? A study on personal rejection was followed by two main studies on vicarious group-based rejection. It was found that merely observing rejection of ingroup members can trigger feelings of humiliation that are equally intense as those experienced in response to personal rejection. Moreover, given that the rejection is explicit, vicariously experienced feelings of humiliation can be accompanied by powerlessness and anger. Potentially, this combination of emotions could be an important source of offensive action against rejecters.

## Introduction

Humiliation is often believed to be a driving force behind social conflicts. Scholars have stressed the role that humiliation plays in, for instance, international politics [1], intractable conflicts [2], genocide [3] psychosocial maladies [4], and high school shootings [5]. The experience of humiliation is typically described as a strong emotional reaction to being ostracized, i.e. to being rejected in the sense of being made to feel small or worthless [6], [7]. In the remainder of this paper, we use ostracism and rejection in this sense synonymously. Humiliation signals to victims that they are rejected in the sense that they are seen as inferior and “not worth bothering with” [8]. For instance, Baumeister, Wotman and Stillwell [9] argued that rejection by a potential romantic partner conveys a symbolic message that one is perceived to lack sufficient desirable qualities to be a worthy partner, and hence induces humiliation. Experiences of social rejection in this sense can create serious deficits in the satisfaction of social needs, with the potential of pathological consequences [10]. Research on emotional and attitudinal responses toward social rejection mostly focuses on situations in which one is personally rejected.

In the present paper, our interest is in rejection and humiliation in intergroup settings, specifically in contexts in which the individual is not personally involved but that concern other ingroup members. The driving question is whether it is possible to feel humiliated by just observing members of one's ingroup being rejected. If people can experience humiliation in response to rejection of others with whom they identify, humiliating experiences could potentially spread easily through a social system. In this article, we examine this possibility of *vicarious* group-based rejection, and the possibility that vicarious rejection triggers emotional responses that are similar to being rejected oneself. Research on humiliation is scarce and to our knowledge, no studies have examined whether people can feel humiliated by just witnessing ingroup members being rejected.

### Humiliation, powerlessness and anger

Central to the experience of humiliation is that one is deprived of power and controlled by a third party [2]. Or, as Lindner [11] puts it, “the victim is forced into passivity, acted upon, made helpless”. Some have suggested that this loss of power may lead to inertia; a tendency toward inaction that suppresses aggressive responses [12] and hence should be accompanied with action-inhibiting emotions like fear and shame. However, in line with appraisal theories of emotion (e.g., [13]) and previous findings on social rejection [14] and humiliation [15], [16] we believe that precisely because the humiliation is inflicted by a perpetrator it is more likely to be associated with action-oriented emotions, predominantly anger [13].

Research findings by Kamans, Otten and Gordijn [17] suggest that when people face an obstacle threat (e.g., to obtaining valued goods), powerlessness is likely to be associated with anger aimed at removing the obstacles, but not with fear. Conversely, although humiliation is often used interchangeably with shame [18], some suggest that the two emotions are likely to be distinct [16]. Whereas humiliation stems from the behavior of others and triggers a focus on the perpetrator, shame is believed to stem from one's own acts and is, therefore, likely to trigger a focus on the self [6], [19]. As such, whereas humiliation is perceived as unfair and undeserved [20], shame signals that one's own behavior can cause exclusion and hence is likely to inhibit behaviors that may potentially cause further damage to social relationships (e.g., aggression). This leads us to propose that feelings of humiliation are likely to be associated with feelings of *powerlessness* and *anger*, and that this also holds for vicarious humiliation.

### Vicarious group-based humiliation: Witnessing rejection of ingroup members

There is some evidence for the similarity of personal and group-membership based experiences. For example, research has shown that being ostracized by a member of an outgroup on the basis of one's group membership triggers levels of distress equal to those triggered by being ostracized as an individual [21], regardless of whether the perpetrator represents one's ingroup, a rival outgroup, or a despised outgroup [22]. We know of only one study in which group-based humiliation was empirically examined. Leidner and colleagues [23] had participants self-identify with a minority group and subsequently recall a situation in which they were humiliated because of their group membership. The authors found that feelings of humiliation are associated with powerlessness and, in some respects, with anger. The proposition we would like to make and test goes one step further. Whereas Leidner et al. [23] examined (memories of) personally experienced group membership-based humiliation, the proposition that is central to our study is that humiliation can also be experienced *vicariously*, i.e., after just observing the rejection of members of the ingroup rather than being personally rejected as an individual or as a member of a group.

A first hint that people may vicariously experience other people's pain stems from empathy research. Focusing mostly on physical pain, research has shown that vicariously experienced pain activates the same brain regions as directly experienced pain and triggers associated responses [24]. In recent years, research on vicarious *social pain* has gradually emerged. Wesselman and colleagues [25] identified nine experimental studies, the majority of which used Williams' [26]–[27] Cyberball paradigm to examine reactions to ostracism in an online ball-tossing game. It was found in these studies that observing ostracism activates relevant brain regions and corresponding feelings in observers (e.g., [28]–[29]). As such, witnessing others being ostracized can make the observer feel ostracized himself. Similar results have been found for vicarious embarrassment [30]. However, empathetic responses to vicarious rejection seem to depend on emotional closeness with the victim [31]–[32]. For example, Wesselman, Bagg and Williams [33] found that observers who consciously identified with a victim of ostracism reported greater need threat than observers who did not. Similarly, Beeney et al. [32] demonstrated that witnessing a friend being included and then excluded prompts empathetic brain activity and that emotional closeness with the victim is a powerful positive moderator of this response.

Supported by existing research findings in social psychology, we suggest that similar results may be expected when the person's relationship with the victim is defined by group membership, such that observing social rejection may trigger feelings of humiliation when the victim belongs to the ingroup, but not to the outgroup. Research has shown that situations that involve (members of) one's ingroup but not oneself can trigger emotional reactions that are similar to those of people who are directly involved. Combining insights from self-categorization theory [34] and appraisal theory [13], [35] Smith [36] developed a model of social emotions based on the idea that when group membership is salient, the ingroup becomes part of the psychological self. In that case, issues that concern the group by definition concern the individual [37]–[38]. As a consequence, people can experience emotions in response to events that affect (members of) their group rather than themselves. For instance, confronting people with ingroup memberś maltreatment of outgroup members induced feelings of guilt in them, even when they personally had not participated in the wrongdoing [39]. Similarly, confronting Surinamese with their group's history of slavery aroused feelings of anger and the desire for reparation by the outgroup [40]. Harth and colleagues [41] examined emotional and attitudinal reactions to rejection of reconciliation gestures by victimized outgroup members. Participants whose ingroup had first transgressed against an outgroup and subsequently offered a reconciliatory gesture (i.e., an apology or repair), reacted with anger (but not shame and anxiety) and offensive responses against the outgroup when the gesture was rejected.

Similar reactions were found for events that did not concern the group as a whole, but individual group members. For example, in research by Gordijn and colleagues [42]–[44], participants observed intentional and unfair behavior by members of an outgroup that negatively affected others, but not themselves. Participants who categorized the victims as belonging to the ingroup experienced more anger than participants who categorized the victims as belonging to the outgroup. Perceiving the victims as ingroup members also led to offensive action tendencies, such as protesting against the perpetrators or trying to prevent the perpetrators' actions. In contrast, participants who perceived the victims as outgroup members reported no anger or offensive action tendencies. Together, these studies demonstrated that people can experience strong emotions when they are not personally involved, but when ingroup members are involved.

In analogy to these findings, we reason that if social rejection in the sense that one is seen as inferior and not worth bothering with can cause feelings of humiliation, and if people can experience emotional responses to events that concern members of their ingroup, then it follows that observing the rejection of ingroup members can activate feelings of humiliation. This possibility has not been well investigated to date. Therefore, in the present study, we aim to test experimentally whether or not vicarious group-based humiliation exists and, if so, whether it equals personal humiliation in strength. Such a finding would be socially relevant, because it would imply that social rejection of a small proportion of a social group might suffice to trigger offensive and action-oriented responses of other group members, aimed at redressing the (vicarious) experience of rejection. Moreover, given that social rejection can cause serious threats to fundamental needs, such as belonging, control, self-esteem, and meaningful existence (e.g., [45]), experiences of humiliation may not only negatively affect individual members, but also the group as a whole.

### The Present Research: Overview and Hypotheses

We conducted a series of experimental studies to test the hypothesis that people can experience humiliation, powerlessness, and anger when they observe that ingroup members are rejected, even when they are not personally involved in the situation. In Study 1, we aimed to show that social rejection is indeed a source of humiliation and, in addition, to establish a paradigm for the vicarious group-based rejection. To that end, we used Williams' [26], [27] Cyberball paradigm as a means to induce personal humiliation. In Study 2, we applied this same paradigm to vicarious group-based rejection and humiliation (our main focus). Study 2 uses a minimal group paradigm that lacks reference to the real world. Therefore, in Study 3, we used existing political differences as basis for ingroup/outgroup differences [22], and we used a stronger form of rejection. In all three studies, we measured feelings of humiliation, powerlessness, and anger after the rejection manipulations. For all our studies, author requirements proposed by Simmons, Nelson and Simonsohn [46] were followed. A minimum of twenty observations per cell was collected, and all observations were collected prior to the data analyses.

## Study 1

The main goal of Study 1 was to test the experimental rejection paradigm for inducing humiliation by examining whether people who are rejected indeed feel humiliated, and whether humiliation is associated with powerlessness and anger. The rejection manipulation was based on Williams' [26], [27] Cyberball paradigm, in which participants believe themselves to be playing a ball-toss game with two or three other players, who are actually computer-programmed confederates. These confederate players are programmed to initially include and then exclude the participant, thereby creating rejection. Because the participants are repeatedly excluded, we can speak of ostracism. Previous research has shown that being ostracized in this game leads people to feel sad and angry, and it lowers levels of belonging, self-esteem, control, and meaningful existence [45]. In this first study, we tested whether it triggered the predicted emotional combination of humiliation, powerlessness, and anger.

### Method

#### Ethics statement

The study and procedures were approved by the Ethical Committee Psychology (ECP) of the Department of Psychology of the University of Groningen. Participants responded to flyers advertising the study, which were distributed in public areas of different university buildings (e.g., the university library, several faculties), and volunteered to participate in the study. Upon arrival, participants were personally welcomed by the researchers and informed about the nature and duration of the study and of any possible risks or difficulties involved. Participants were explicitly told that participation in the study was anonymous and voluntary and that they could decide to withdraw at any moment, or could choose to refrain from answering questions in the survey without consequences of any type. When participants decided to participate in the study, they received the same information again on the introduction screen of the study. All data were anonymized upon collection by assigning each participant with a unique identifier, which does not link to the participant's identity. After the study, the participants were personally debriefed in writing (i.e., on the screen) as well as orally by the researchers, during which they were again informed that they could decide to withdraw their answers. No participants objected to having their data analyzed.

#### Participants and design

Thirty-five male and 24 female undergraduate students (*M*age  = 21.80, *SD* = 2.65) from the University of Groningen participated voluntarily in the study in exchange for 5 euros. Participants were randomly assigned to one of two treatments: being ostracized or included in a game of Cyberball that lasted for 30 tosses [27]. Based on a discussion in the literature that the experience of humiliation might be intensified by the presence of an audience that witnesses the rejection [6], [15] we included a between-subjects factor (exposure: public vs. private) to explore whether the sensation of being publicly exposed enhanced humiliation after rejection. In half of the cases we attached a webcam to the screen and told participants in the introduction to the study that the interaction would be observed by a group of psychology students, who were allegedly taking a practicum in another room in the building. In reality, the webcam was not connected and no footage was recorded. As our focus was on the link between rejection and humiliation rather than on the role of public exposure, and the presence of a webcam did not influence the results (multivariate *p* = .56), we decided not to include the factor in the analyses.

#### Procedure

Upon arrival at the testing room, participants were seated at computers in individual cubicles. All instructions were presented on the screen. Participants were asked to log on to Cyberball with two other (computer-generated) participants. Depending on the condition, participants received one third of the tosses (inclusion condition), or only two and then none for the remainder of the game (ostracism condition). Throughout the game, every player was represented by an icon on the screen that was identified by the player's initials.

#### Dependent variables

After the game, participants were asked to rate on a series of 7-point scales ranging from 1 (absolutely not) to 7 (absolutely) how they had felt during the game. We measured to what extent the participants had felt *humiliated* (i.e., degraded, humiliated, belittled; Cronbach's *α* = .91), *powerless* (i.e., impotent, helpless, discouraged; Cronbach's *α* = .82), and *angry* (i.e., angry, outraged, annoyed; Cronbach's *α* = .87). We also measured shame (i.e., ashamed, feeling shame; Cronbach's *α* = .91) and fear (i.e., anxious, afraid, scared; Cronbach's *α* = .73). We also measured the extent to which they felt *happy* (i.e., optimistic, happy, cheerful, positive; Cronbach's *α* = .90). To avoid the impression that we expected participants to have particular emotions, the items were collected as part of a larger battery of twenty-four emotions. In addition, in order to increase the salience of ingroup/outgroup differences, some questions were asked about how the participants believed they were being viewed by the other players in the game, and about their ideas about what kind of people the other players were. These extra items by themselves were of no interest to us, other than their function to improve the measurement of the focal variables. We therefore do not discuss them further in this paper.

### Results

#### Humiliation

We tested our predictions by performing an ANOVA using the ostracism manipulation (inclusion vs. ostracism) as the predictor of feelings of humiliation. As expected, humiliation was significantly predicted by the ostracism manipulation, *F* (1, 57) = 17.16, *p<*.01, *η_p_^2^* = .23. Being ostracized triggered stronger feelings of humiliation (*M* = 3.24, *SD* = 1.51) than being included (*M* = 1.77, *SD* = 1.12).

#### Powerlessness

The ostracism manipulation also had a significant effect on feelings of powerlessness, *F* (1, 57) = 10.35, *p<*.01, *η_p_^2^* = .15. Ostracized participants felt more powerless (*M* = 3.14, *SD* = 1.22) than included participants (*M* = 2.08, *SD* = 1.29).

#### Anger

Also as expected, the ostracism manipulation significantly predicted feelings of *anger, F* (1, 57) = 11.85, *p<*.01, *η_p_^2^* = .17. Ostracized participants experienced more anger (*M* = 3.24, *SD* = 1.51) than included participants (*M* = 1.97, *SD* = 1.33).

#### Other emotions

With respect to *fear* (*M* = 1.63, *SD* = 0.83) and *shame* (*M* = 1.77, *SD* = 1.03), no significant effect of ostracism was found, *F*’s*<*1. With respect to *happiness,* a significant effect of the ostracism manipulation was found showing that people in the inclusion condition felt happier (*M* = 4.63, *SD* = 1.19) than people in the ostracism condition (*M* = 3.63, *SD* = 1.01), *F* (1, 57) = 11.86, *p<*.01, *η_p_^2^* = .17.

#### Factor analysis

To examine the proposed link between humiliation, powerlessness and anger, we performed a factor analysis. The 18 items were submitted to a principal component analysis (Varimax rotation with Kaiser Normalization). The factor solution revealed 3 factors with an Eigenvalue > 1. As can be seen in Table 1, we found the expected humiliation factor with the humiliation, powerlessness, and anger items (Eigenvalue  = 9.15; explained variance  = 51%). Further, we found a ‘happiness' factor that included all positive items (Eigenvalue  = 1.30; explained variance  = 7%), and a factor that included the shame and fear items (Eigenvalue  = 2.42; explained variance  = 13%).

**Table 1:** Rotated structure matrix: Study 1.

	Humiliation	Shame and	Happiness
		Fear	
Humiliated	**.791**	.230	.318
Degraded	**.809**	.154	.315
Belittled	**.774**	.225	.374
Impotent	**.795**	.297	−.006
Helpless	**.672**	.439	.223
Discouraged	**.569**	.483	.455
Outraged	**.784**	.269	.307
Angry	**.839**	.155	.271
Annoyed	.*545*	.313	.*558*
Anxious	.057	**.685**	.054
Scared	.134	**.796**	.314
Afraid	.234	**.748**	−.009
Ashamed	.433	**.778**	.042
Feeling shame	.318	**.764**	−.087
Optimistic	−.344	.121	**−.602**
Happy	−.075	−.039	**−.861**
Positive	−.402	−.191	**−.670**
Cheerful	−.251	−.066	**−.877**

*Note*. Extraction method: Principal component analysis. Rotation method: Varimax with Kaiser Normalization.

### Discussion

The findings of Study 1 suggest that people can feel humiliated after being ostracized in the Cyberball paradigm. As such, Study 1 offers a useful starting point to move from examining the link between personal rejection and humiliation to examining that between vicarious group-based rejection and humiliation, which was the central focus of the present research. Study 1 replicated earlier findings on social rejection (for a review, see [48], [49]) and on the characteristic link of humiliation with both powerlessness and anger [23]. That humiliation does not necessarily lead to inertia (as suggested by [12]) can be gleaned from the fact that factor analysis revealed the combination of humiliation, powerlessness, and anger as a separate construct that was distinct from happiness as well as from shame and fear. Moreover, social rejection had no influence on shame and fear, which are both emotions that tend to inhibit action.

## Study 2

In Study 2 we used the research paradigm of Study 1 to test our proposition that vicarious group-based rejection can cause feelings of humiliation. In addition to a condition in which subjects were personally rejected, we included a condition in which subjects observed other ingroup members being rejected by members of a salient outgroup. Previous research by Gonsalkorale and Williams [22] has shown that even if people are rejected by members of a despised outgroup (e.g., the KKK) they show strong rejection effects. Furthermore, research by Gordijn and colleagues [42]–[44] suggests that people are likely to respond emotionally to observing others being victimized when the victims belong to the ingroup, but not when they belong to the outgroup. Thus, we predicted that vicarious group-based humiliation is mainly ingroup-based, meaning that it is more strongly triggered when members of an outgroup reject members of the ingroup than when they reject members of an outgroup.

### Method

#### Ethics statement

The study and procedures were approved by the ethics committee of the Department of Sociology of the University of Groningen. Whereas Studies 1 and 3 were approved by the Ethical Committee Psychology (ECP) of the Department of Psychology of the University of Groningen, Study 2 was authorized by the Ethical Committee of the Department of Sociology, because for this study we only recruited undergraduate sociology students. Written consent was obtained. Prior to the study, participants were informed orally by the researchers about the duration and nature of the study. Participants were told that participation was anonymous and voluntary and that they could decide to withdraw at any moment or choose to refrain from answering questions in the survey without consequences of any type. The same information was also provided on paper on the introduction page of the questionnaire. All data were anonymized upon collection by assigning each participant with a unique identifier, which does not link to the participant's identity. After the study, the participants were personally debriefed in writing (i.e., on the screen) as well as orally by the researchers.

#### Participants and design

Twenty-nine male and 45 female undergraduate sociology students from the University of Groningen participated in the experiment in exchange for 1 study credit (*M*age  = 20.09, *SD* = 1.58). Participants were randomly assigned to one of only three conditions in the Cyberball paradigm: 1) a “personal rejection” condition, in which the participants were themselves ostracized by two members of an outgroup; 2) a “vicarious ingroup rejection” condition, in which they observed one ingroup member being ostracized by two outgroup members, or 3) a “vicarious outgroup rejection” condition, in which they observed one outgroup member being ostracized by two members of yet another outgroup.

#### Procedure

As in Study 1, participants were seated at a computer in individual cubicles and the instructions were presented on the screen. In the personal rejection condition, the instructions were copied from Study 1. In the two vicarious rejection conditions, participants were told that it was being investigated how outsiders perceived social interactions among groups of people and that they would observe an online ball-tossing game (Cyberball: [27]) between other participants (who were actually computer-generated confederates).

The participants then filled in a political preference test, on the basis of which they were allocated to one of three groups: the “Leftish”, “Rightish”, or “Central” group. Subsequently, the participants played in or observed a three-player Cyberball game in which two perpetrators, who always shared the same political preference, rejected the third player. The perpetrators' identities depended on the participants own identities. For leftish participants, the perpetrators always represented the rightish group, and vice versa. For central participants, the perpetrator also represented the rightish group, because the sample was predominantly left-oriented (84%). This implies that a) in the personal rejection condition, both leftish and central participants were rejected by rightish perpetrators, whereas rightish participants were rejected by leftish perpetrators; b) in the vicarious ingroup rejection condition, leftish and central observers witnessed one ingroup member being rejected by two rightish outgroup members, whereas rightish observers witnessed one ingroup member being rejected by two central outgroup members; and c) in the vicarious outgroup rejection condition, leftish and rightish participants observed two central players reject one rightish or leftish player, respectively, whereas central players observed two leftish players reject one rightish player. After the game the same dependent measures were taken as in Study 1.

### Results

#### Humiliation

**Figure 1:**
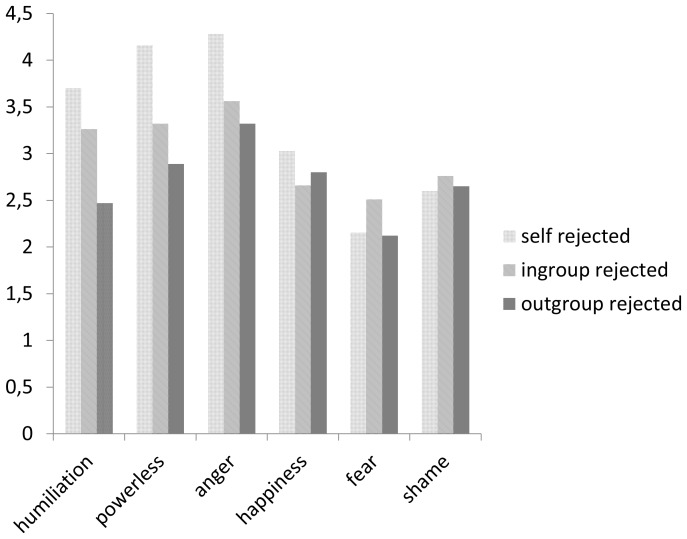
Dependent measures as a function of rejection of self, ingroup, or outgroup (Study 2).

We tested our predictions by performing oneway ANOVAs using the ostracism manipulation (self, ingroup, or outgroup) as the predictor of feelings of humiliation. As expected, humiliation was significantly predicted by the ostracism manipulation, *F* (2, 71) = 4.62, *p<*.05, *η_p_^2^* = .12. More humiliation was experienced when participants were ostracized (*M* = 3.70, *SD* = 1.52) or when members of their ingroup were ostracized (*M* = 3.26, *SD* = 1.42), than when members of an outgroup were ostracized (*M* = 2.47, *SD* = 1.28). The difference between personal rejection and rejection of ingroup members by the outgroup was not significant, as shown by least significant differences tests (*p* = .29). Both of these conditions differed significantly from the condition in which outgroup members were rejected (*p* = .004 and *p* = .043, respectively). This was in line with the hypotheses on vicarious group-based humiliation; see Figure 1.

#### Powerlessness

The ostracism manipulation also had a significant effect on feelings of powerlessness, *F* (2, 71) = 5.69, *p<*.01, *η_p_^2^* = .14, but the personal rejection had a stronger effect than the vicarious rejection. When the effects of personal rejection were compared with the effects of rejection of ingroup members by the outgroup, a least significant differences test showed that feelings of powerlessness were significantly higher after personal rejection (*M* = 4.17, *SD* = 1.21) than after rejection of ingroup members (*p* = .027), or than after rejection of outgroup members by members of another outgroup (*p* = .001). We did find that more powerlessness was experienced when outgroup members rejected ingroup members (*M* = 3.32, *SD* = 1.19), than when they rejected outgroup members (*M* = 2.89, *SD* = 1.40). However, this difference did not reach significance (*p* = .22).

#### Anger

The ostracism manipulation produced a marginally significant effect on *anger, F* (2, 71) = 2.74, *p<*.07, *η_p_^2^* = .07. A least significant differences test showed that anger in response to personal rejection (*M* = 4.28, *SD* = 1.02) was of similar strength to anger in response to vicarious ingroup rejection (*M* = 3.56, *SD* = 1.44; *p* = .09), and significantly stronger than in response to vicarious outgroup rejection (*M* = 3.32, *SD* = 1.64; *p* = .026). However, no significant differences were found between vicarious rejection of the ingroup and of the outgroup (*p* = .54). Therefore, the hypothesis that vicarious ingroup rejection triggers more anger than vicarious outgroup rejection is not supported by the data.

#### Other emotions

With respect to *happiness, sham,* and *fear,* no significant differences were found; all *F*s*<*1.

#### Factor analysis

As in Study 1, we examined using a factor analysis whether feelings of humiliation were more closely related to feelings of anger and powerlessness than to happiness, fear, and shame. The 18 items were subjected to a principal component analysis. We performed a Varimax rotation with Kaiser Normalization. The results were relatively similar to those of Study 1. The factor solution revealed 3 factors with an Eigenvalue > 1. As can be seen in Table 2, we found the expected humiliation factor with the humiliation, powerlessness, and anger items (Eigenvalue  = 6.64; explained variance  = 37%), although two powerlessness items (impotent and helpless) also loaded on the ‘fear and shame’ factor that contained the shame and fear items (Eigenvalue  = 3.11; explained variance  = 17%). Further, we found a ‘happiness' factor that included all positive items (Eigenvalue  = 1.72; explained variance  = 10%).

**Table 2:** Rotated structure matrix: Study 2.

	Humiliation	Shame and	Happiness
		Fear	
Humiliated	**.761**	.169	−.010
Degraded	**.770**	.071	.066
Belittled	**.823**	.180	−.041
Impotent	.*510*	.478	.047
Helpless	.*485*	.*514*	−.093
Discouraged	**.712**	.283	−.142
Outraged	**.712**	.190	.030
Angry	**.604**	.366	−.075
Annoyed	**.814**	.257	−.081
Anxious	.252	**.724**	−.050
Scared	.161	**.823**	.026
Afraid	.246	**.811**	.051
Ashamed	.320	**.711**	.010
Feeling shame	.094	**.663**	.037
Optimistic	−.001	−.083	**.868**
Happy	−.026	.146	**.810**
Positive	−.047	−.052	**.915**
Cheerful	−.051	.029	**.893**

*Note*. Extraction method: Principal component analysis. Rotation method: Varimax with Kaiser Normalization.

### Discussion

The results support the conjecture that humiliation may also be vicarious and group-based. Participants who had observed rejection of an ingroup member felt as humiliated as participants who were personally rejected and significantly more humiliated than participants who observed the rejection of an outgroup member. This suggests that vicarious humiliation may be confined to what happens to members of the ingroup.

Findings with respect to powerlessness and anger were more ambiguous. On the one hand, personal rejection produced more powerlessness and anger than vicarious rejection of outgroup members, which is in line with our hypothesized combination of humiliation, powerlessness, and anger. On the other hand, we found no support for the hypothesis that vicarious ingroup rejection triggers more powerlessness and anger than does vicarious outgroup rejection: participants felt equally powerless and angry in both conditions. Also, the factor analysis suggests that at least an element of powerlessness is closely associated with shame and fear, which suggests an action-inhibiting response. Together, the findings suggest that indeed both personal and vicarious forms of rejection are accompanied by humiliation. However, for effects on powerlessness and anger, the humiliation was possibly too indirect and the difference between ingroup and outgroup was possibly too small in Study 2. Therefore, in Study 3, we made some adaptations to the design.

## Study 3

In order to increase the difference between ingroup and outgroup and the strength of humiliation, in Study 3 we used ideologically diverse social groups to manipulate an intergroup context and made humiliation more direct. To prevent emotional responses from being confounded by the (numerical) minority status of the victim, in Study 3 we balanced the numbers of perpetrators and victims. Whereas we relied on the Cyberball paradigm [27] for the ostracism manipulation in the previous two studies, in Study 3 we applied a procedure in which participants were first included and in the next round explicitly rejected, or in which they watched others being rejected in this way (for similar procedures, see [47], ). Specifically, participants were told that the perpetrators did not consider them worth interacting with. We hypothesized that both personal rejection and observed rejection of ingroup members would trigger higher levels of humiliation, anger, and powerlessness than observed rejection of outgroup members or no rejection. Due to the mixed results of Study 2 and the extended duration of Study 3 (see below), following our attempts to improve the research design, we decided to prioritize our aim of testing the combination of vicariously experienced humiliation, powerlessness, and anger (the focus of our study) at the expense of a test to disentangle these emotions from action-inhibiting emotions. Consequently, given time constraints and to avoid conducting an unreasonably lengthy experiment, we dropped the measures for fear and shame. We included a control condition without rejection in order to see whether observing an outgroup being rejected by an outgroup creates effects that are any different from no rejection at all.

### Method

#### Ethics statement

Same as in Study 1.

#### Participants and design

Sixty-seven male and 30 female undergraduates of the University of Groningen participated in the experiment in exchange for 5 euros (*M*age  = 19.33, *SD* = 1.25). To manipulate social rejection, participants were randomly assigned to one of only three experimental conditions in which they were told that other participants in the experiment (perpetrators) did not want to continue playing a game with two other players (victims) because they perceived these players not worth interacting with: 1) a “personal rejection” condition; 2) a “vicarious ingroup rejection” condition; 3) a “vicarious outgroup rejection” condition. Moreover, a control condition was included in which no social rejection occurred.

#### Procedure

Participants were seated at computers in individual cubicles. All instructions were presented on the screen. The participants were informed that the researchers were examining group performance and that they would play or observe an online ball-game followed by an online quiz with other participants, who would be logged on at the same time. Based on the results of a political preference test, participants were then allocated to one of three groups that represented different political views: the “Socialist” group, the “Liberal” group, or the “Conservative” group. To keep group-affiliation salient, we coupled participants' initials with the name of their ingroup and addressed them accordingly throughout the experiment. After being assigned to a group, participants were informed that four participants would be randomly selected to play the ball-game and the quiz, and that the remaining participants would be selected as observers. Subsequently, the participants played or observed the ball-game, which served as a filler task before the manipulation to strengthen participants' group identity.

After the ball-game, the ‘players' were seemingly given the option of playing the quiz in a homogenous team of players who shared their political preference, or in a heterogeneous team of players with different political preferences. Their answers appeared on the screen, and represented the social rejection manipulation. In the three rejection conditions, two players who shared political preferences expressed no interest in teaming up with players with different political views, whom they perceived as inferior to themselves. Specifically, one of them claimed that *“I do not want to be associated with these two {ingroup/outgroup} people. I don’t understand how people can be so foolish as to adhere to such worthless ideologies and I would be embarrassed if I had to work together with them. It*'*s just best to ignore them”.* In line with this, the other player said: *“I have no interest in working with the players of the {Ingroup/Outgroup}. These people have a very narrow minded worldview; you could tell that even from the ball-game we just played. I do not want to work together with them”*. For players in the personal rejection condition, this meant that they were personally rejected. Participants in the two vicarious rejection conditions witnessed (as observers) the rejection of two members of either their ingroup or an outgroup by members of a third outgroup. Participants in the control condition were informed that the players for the quiz had been randomly selected. The players in that condition represented two ingroup members and two outgroup members; no rejection statements were made.

As in Study 2, the identities of the players depended on the participants' own identities. For socialist and liberal participants, the perpetrators always represented the conservative group; for conservative players, the perpetrators always represented the socialist group. We decided on this allocation because we assumed that both liberal and socialist participants would perceive the conservative group as the most ideologically distant from the ingroup. We tested this assumption using a pre-measure of participants' most and least preferred ideological perspectives. Of the 38 participants who identified most with the socialist perspective, 32 disliked the conservative views most. Similarly, of the 49 participants who identified most with the liberal viewpoint, 35 ticked the conservative view as their least favorite political perspective. This allocation rule implies that a) in the personal rejection condition both socialist and liberal participants were rejected by conservative perpetrators, whereas conservative participants were rejected by socialist participants; b) in the vicarious ingroup rejection condition, socialist and liberal participants witnessed two ingroup members being rejected by two conservative outgroup members, whereas conservative observers witnessed the rejection of ingroup members by socialist outgroup members; c) in the vicarious outgroup condition, socialist and liberal participants observed two conservative players reject two liberal and socialist players, respectively, whereas conservative players observed two socialist players reject two liberal players. After this, we measured feelings of humiliation, powerlessness, anger, and happiness as in the first two studies.

### Results

#### Humiliation

**Figure 2:**
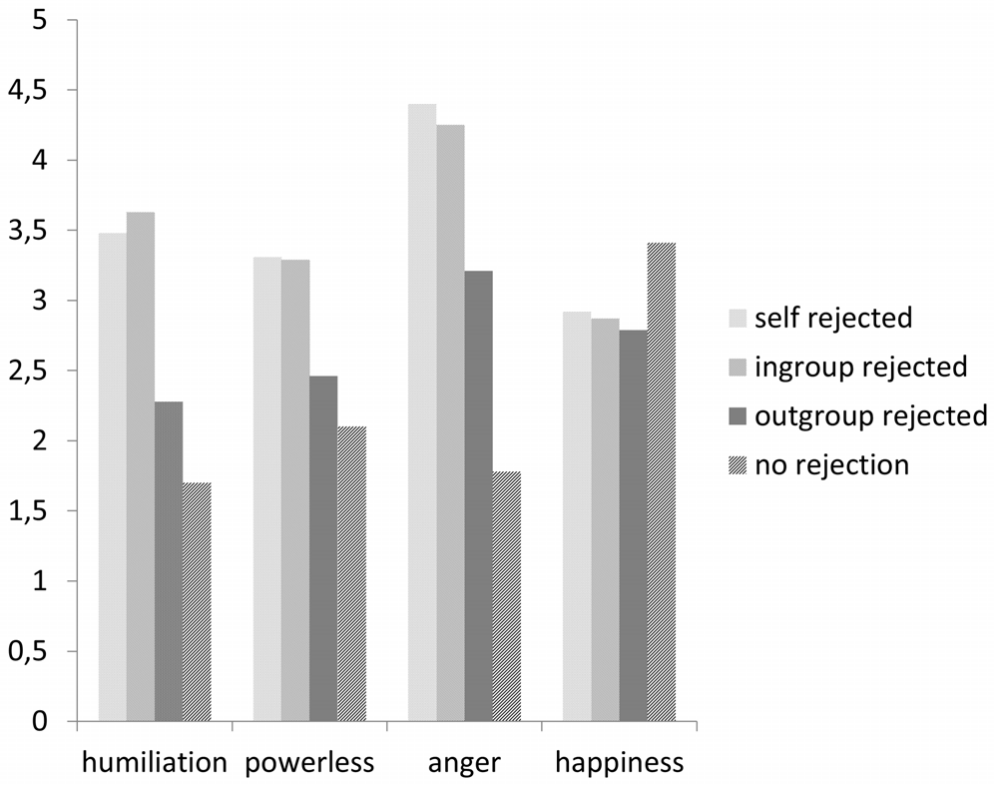
Dependent measures as a function of rejection of self, ingroup, or outgroup, or no rejection (Study 3).

We tested our predictions by performing oneway ANOVAs with the social rejection manipulation as the predictor of feelings of humiliation. As expected, humiliation was significantly predicted by the rejection manipulation, *F* (3, 93) = 11.91, *p<*.01, *η_p_^2^* = .28. Participants experienced more humiliation when they were personally rejected (*M* = 3.48, *SD* = 1.69) or when members of their ingroup were rejected (*M* = 3.63, *SD* = 1.30), than when members of an outgroup were rejected (*M* = 2.28, *SD* = 1.21), or when no rejection occurred (*M* = 1.70, *SD* = .98). Least significant differences tests showed that the personal rejection and ingroup rejection conditions did not differ from each other (*p* = .70), whereas they both differed from the outgroup rejection condition (both *p*s <.01), and the control condition (both *p*s*<*.01).The outgroup rejection condition and the control condition did not differ significantly (*p* = .14); see Figure 2.

#### Powerlessness

Supporting our hypothesis, the rejection manipulation also had a significant effect on feelings of powerlessness, *F* (3, 93) = 5.58, *p<*.01, *η_p_^2^* = .15. Participants felt more powerless when they were rejected (*M* = 3.31, *SD* = 1.49), or when members of their ingroup were rejected (*M* = 3.29, *SD* = 0.99), than when members of an outgroup were rejected (*M* = 2.46, *SD* = 1.34), or when no rejection occurred (*M* = 2.10, *SD* = 1.16). Least significant differences tests showed that the personal rejection and ingroup rejection conditions did not differ from each other (*p* = .97), whereas they both differed from the outgroup rejection condition (both *p*s*<*.05), and the control condition (both *p*s*<*.01). The outgroup rejection condition and the control condition did not differ significantly (*p* = .33).

#### Anger

Also as expected, the rejection manipulation significantly predicted feelings of *anger, F* (3, 93) = 18.71, *p<*.01, *η_p_^2^* = .38. Participants felt angrier when they were rejected (*M* = 4.40, *SD* = 1.46), or when members of their ingroup were rejected (*M* = 4.25, *SD* = 1.17), than when members of an outgroup were rejected (*M* = 3.21, *SD* = 1.68), or when no rejection occurred (*M* = 1.78, *SD* = 1.03). Least significant differences tests showed that the personal rejection and ingroup rejection conditions did not differ from each other (*p* = .70), whereas they both differed from the outgroup rejection condition (both *p*s*<*.01) and the control condition (both *p*s*<*.01). The outgroup rejection condition and the control condition also differed significantly (*p<*.01), suggesting that observing outgroup members being unfairly rejected does make people somewhat angry (but less so than when the self or ingroup members are rejected).

#### Happiness

No significant differences were found with respect to *happiness*, *F<*1, *p* = .48. See Figure 2 for all means.

### Discussion

In line with our hypotheses, these results indicate that being rejected by members of an outgroup triggers a combination of humiliation, powerlessness, and anger. In this study we used a stronger (more direct) rejection manipulation and more pronounced ingroup/outgroup differences. Rather than being subtly ostracized in a Cyberball game (as in Studies 1 and 2), participants learned in this study that members of other groups did not want to team up with the players from their group (explicit rejection). Whereas Study 2 produced ambiguous results pertaining to vicarious group-based powerlessness and anger, the findings in Study 3 (using a stronger rejection manipulation) were unambiguously in line with the hypothesis that people can experience humiliation vicariously after observing ingroup members (but not outgroup members) being rejected, and that humiliation is accompanied by powerlessness and anger. In fact, the results suggest that the intensity of emotional responses to the rejection of ingroup members can be equal to that of emotional responses to being personally rejected. In sharp contrast, observing the rejection of outgroup members did not evoke much stronger emotional responses than observing no rejection at all.

## General Discussion

Although humiliation is often mentioned as a source of violent conflict, including genocide [7] and terrorism [52], the experience of humiliation is still poorly understood [16]. Of particular importance for the social relevance of humiliation is the possibility that it can be experienced vicariously, after observing ingroup members being rejected, even when one is not personally involved in the event. We argued and found that being rejected or seeing ingroup members being rejected as inferior and/or not worth bothering with, triggers the experience of being humiliated. We also argued and found that (vicarious) experiences of humiliation are accompanied by feelings of powerlessness and anger, which fits with previous findings and ideas by scholars in the field of humiliation [5−7], [15−16], .

As far as we know, the consequences of vicarious group-based rejection had not been investigated previously (see also [25]). Some research emerged recently on vicarious ostracism [31]–[32]. For instance, Wesselman, Bagg, and Williams [33] found that simply observing ostracism causes negative affect and threats to fundamental needs. However, the authors did not examine the role of group membership. We predicted that only the rejection of *ingroup* members would trigger the combination of humiliation, powerlessness, and anger. This combination of humiliation, powerlessness and anger found support in our studies, which links humiliation to action oriented emotions. Moreover, although the findings of Study 2 were ambiguous regarding the ingroup-based effect for powerlessness and anger (but not humiliation), the findings of Study 3 were unambiguously in line with the hypothesis that vicariously experienced humiliation is confined to ingroup members only.

Remarkably, the findings of Study 3 suggest that people can experience vicarious group-based humiliation as intensely as personal humiliation. Moreover, the findings of Studies 1 and 2 offer preliminary indications that humiliation may be more likely to be associated with action-oriented emotions such as anger than with action-inhibiting emotions such as fear and shame. Although further data are required to corroborate these findings, such effects do point at the potential importance of vicarious group-based humiliation. Without being targeted personally, people can experience negative rejection effects (e.g., needs deficits, pathological consequences [27]) just by observing their ingroup being rejected. And due to the links between humiliation and anger and, in turn, between anger and aggressive action tendencies [14], the rejection of only a few group members may suffice to trigger anger and aggressive responses from entire groups who respond emotionally to the plight suffered by those with whom they identify. In other words, due to vicarious humiliation effects, aggressive reactions might spread quite easily.

Smith's model of social emotions [36] adds theoretical credence to such potential implications. Smith argues that when group membership is salient, the ingroup becomes part of the psychological self, resulting in the experience of emotions in response to events affecting (members of) the ingroup. Previous research supporting that model has revealed that people can experience group-based anger [44], group-based guilt [39], and group-based fear [53]. In line with those findings, in the present research, especially in Study 3, we found preliminary support for not only group-based anger but also group-based feelings of humiliation and powerlessness. Together, these findings add to the literature on both group-based emotions and social rejection and create a launch pad for further investigations of the possibility of vicarious group-based humiliation.

### Future research

Although humiliation is receiving increasing empirical attention (e.g., [23]), little is known about the causes and exact dimensions of the experience. We focused on the role of social rejection, but further research is needed to narrow down the exact conditions under which people feel humiliated. For example, we propose that social rejection in the sense of being made to feel worthless can cause humiliation. It would be interesting to compare this rejection effect with lowering people's power or taking away their experience of control. Would they show emotional responses that are similar to those in response to being rejected? Further, the scholarly debate on humiliation would benefit from an analysis of the relation between humiliation and related emotions such as fear and, in particular, shame. The literature is inconclusive as to whether and how humiliation and shame are connected, although the general consensus appears to be that although similar in some respects, they have distinct features and produce different behavioral responses (e.g., [16]). In our study, humiliation was not closely related to shame, although some elements of powerlessness, which we believed to be part of the humiliating experience, appeared to be connected with shame and fear. Moreover, Leidner et al. [23] found some overlap between humiliation, anger, and shame, which led them to conclude that “the emotional experience of ‘‘humiliation’’ is like that of ‘‘anger’’ in some respects, and like ‘‘shame’’ in others, but it is not the same as either one” (p4).

Future research may also examine specific behavioral responses related to the combination of humiliation, powerlessness, and anger. This is potentially a dangerous mix. For example, research by Kamans, Otten, and Gordijn [17] revealed that group members who feel powerless and angry are more likely to confront an outgroup in reaction to a threat, especially if they strongly identify with their ingroup. Further, research by Kamans, Otten, Gordijn, and Spears [54] has shown that people who perceive their ingroup as powerless are more likely to show unconstructive behavior (e.g., use of threat or demands, negative tone and/or abusive language) in intergroup conflict when their position seems to be hopeless. Combined with humiliation, such offensive reactions might be even stronger.

Studies 1 and 2 were conducted among minimal groups. In Study 3, we used existing political differences to define groups. We saw that the results were more pronounced when we used real-life differences between the groups. Still, even though we observed the predicted differences between conditions, in absolute terms, humiliation experienced by the subjects was not strong. It stands to reason that experiences of vicarious group-based humiliation are intensified when group membership is real or permanent rather than temporary. For example, Wirth and Williams [21] found that participants with temporary, permanent, or no group membership initially felt equally distressed after being ostracized, but that permanent group membership caused slower recovery than temporary group membership. A step further in this direction would be to investigate (vicarious) humiliation in real-life intergroup contexts, such as the Palestinian/Israeli conflict. Compared with research conducted in the laboratory, studies of real-life experiences of humiliation might also find a more pronounced negative effect on feelings of happiness and joy, as was found by Ginges and Atran among Palestinians [12].

It would also be relevant to examine whether emotional responses of humiliation, powerlessness, and anger to vicarious rejection of ingroup members are limited to situations in which members of the ingroup are rejected by outgroup members (the focus of the present study), or can also be experienced when ingroup victims are rejected by ingroup perpetrators. On the one hand, research findings (e.g., [55]) suggest that people may be more sensitive to rebuffs from outgroup members than from ingroup members, such that rejection of ingroup members by other ingroup members might be tolerated relatively well. On the other hand, the black sheep effect [56] suggests that threatening ingroup members may be judged negatively, which would lead to the derogation and rejection of ingroup members who humiliate other ingroup members. Future research may investigate the role of factors such as identification with the group and the conditions under which the humiliation takes place to further examine the dimensions of vicarious group-based humiliation.

### Limitations of the present study

Although the present study has yielded some interesting findings, its design is not without flaws. Several caveats deserve mentioning. A major limitation of the present research is that several confounding factors exist, which were not measured in this study but which may influence the observed outcomes. For example, given that the experience of humiliation is likely to encompass a sense of being lowered and being made to feel inferior, it would have been relevant to explicitly measure the extent to which participants actually experienced this lowering of status and whether they perceive the perpetrators as superior to themselves. Control and the intensity of powerlessness may also have played a role. Participants in the present study lacked opportunities to respond to the rejection (e.g., by reacting to the rejection or directly addressing the perpetrators); this lack of control may have intensified emotional responses of powerlessness.

Also, in Study 3, a rejection manipulation was used in which participants were explicitly rejected on the basis of their group membership, which was in turn based on political preferences. It is not known to what degree rejection on the grounds of political preferences is experienced as both personal and group-based rejection. This too would be worth-while investigating in future research.

In sum, although we believe that our findings provide an encouraging starting point for further research on vicarious humiliation, more research, also outside the laboratory, is needed to clearly delineate the conditions for vicarious humiliation, to disentangle the concept of humiliation from other emotions, and to examine the potential behavioral outcomes of (vicarious) humiliation. What we did show is that vicarious group-based humiliation can be substantial. It therefore is worth receiving much more attention than it has had so far.
